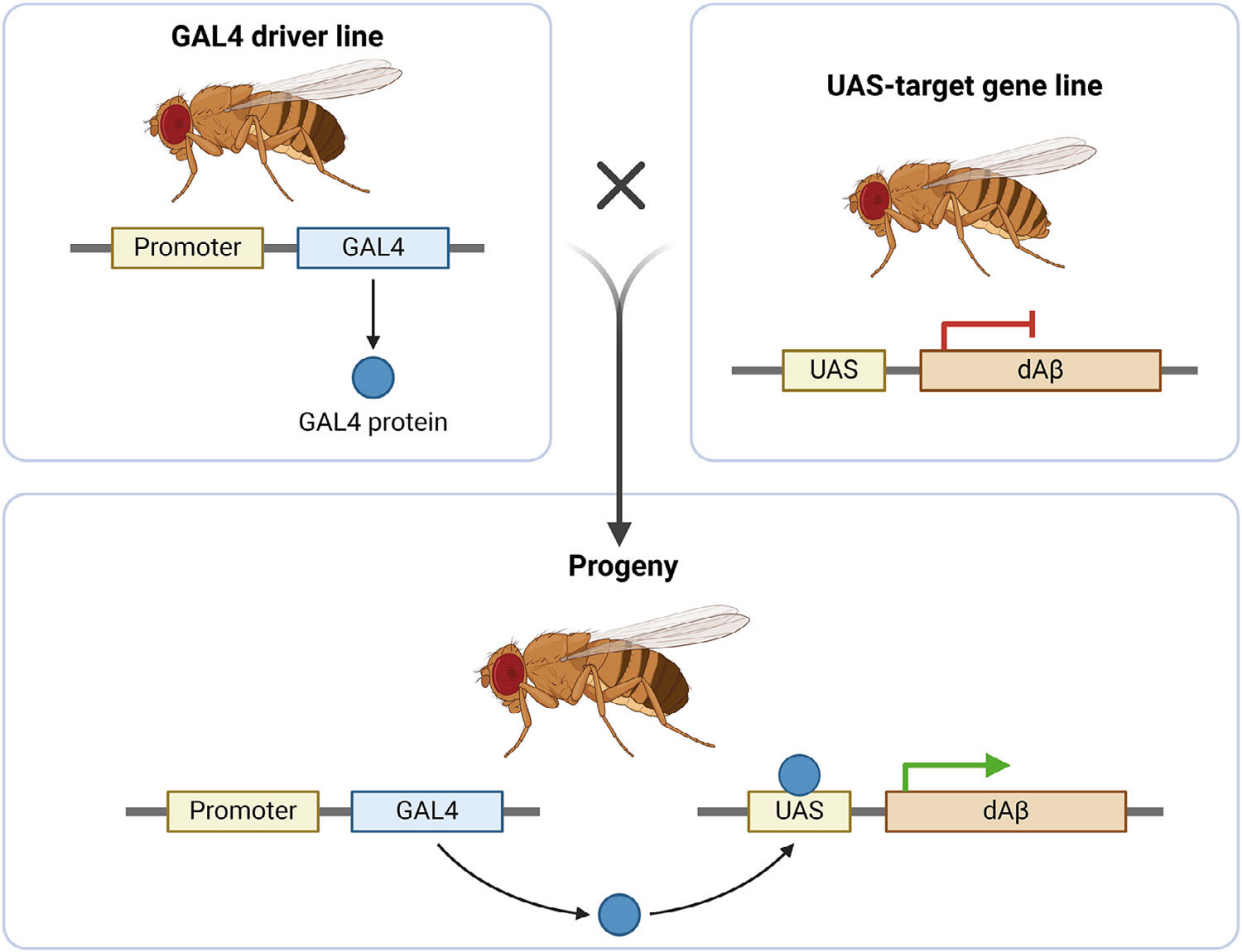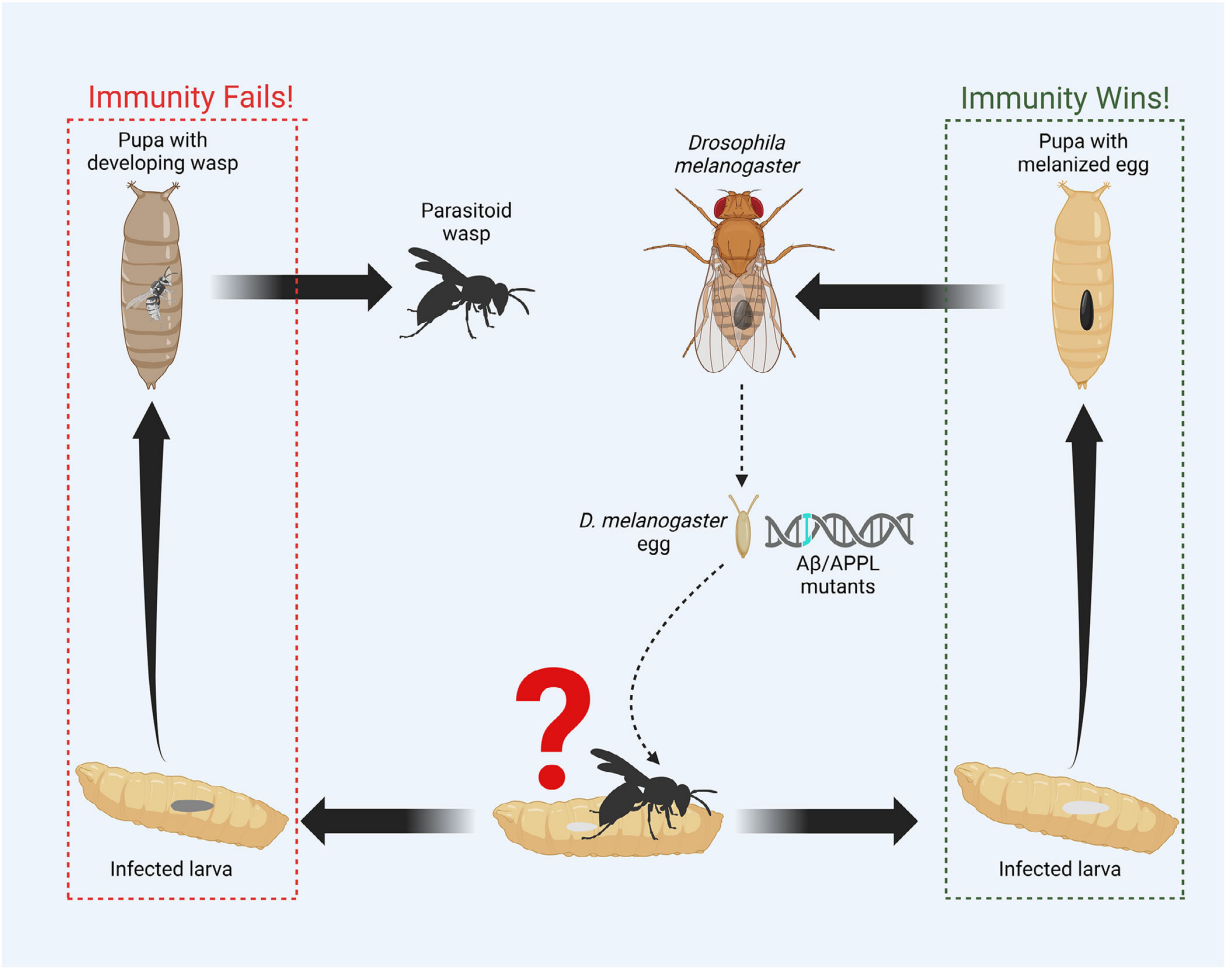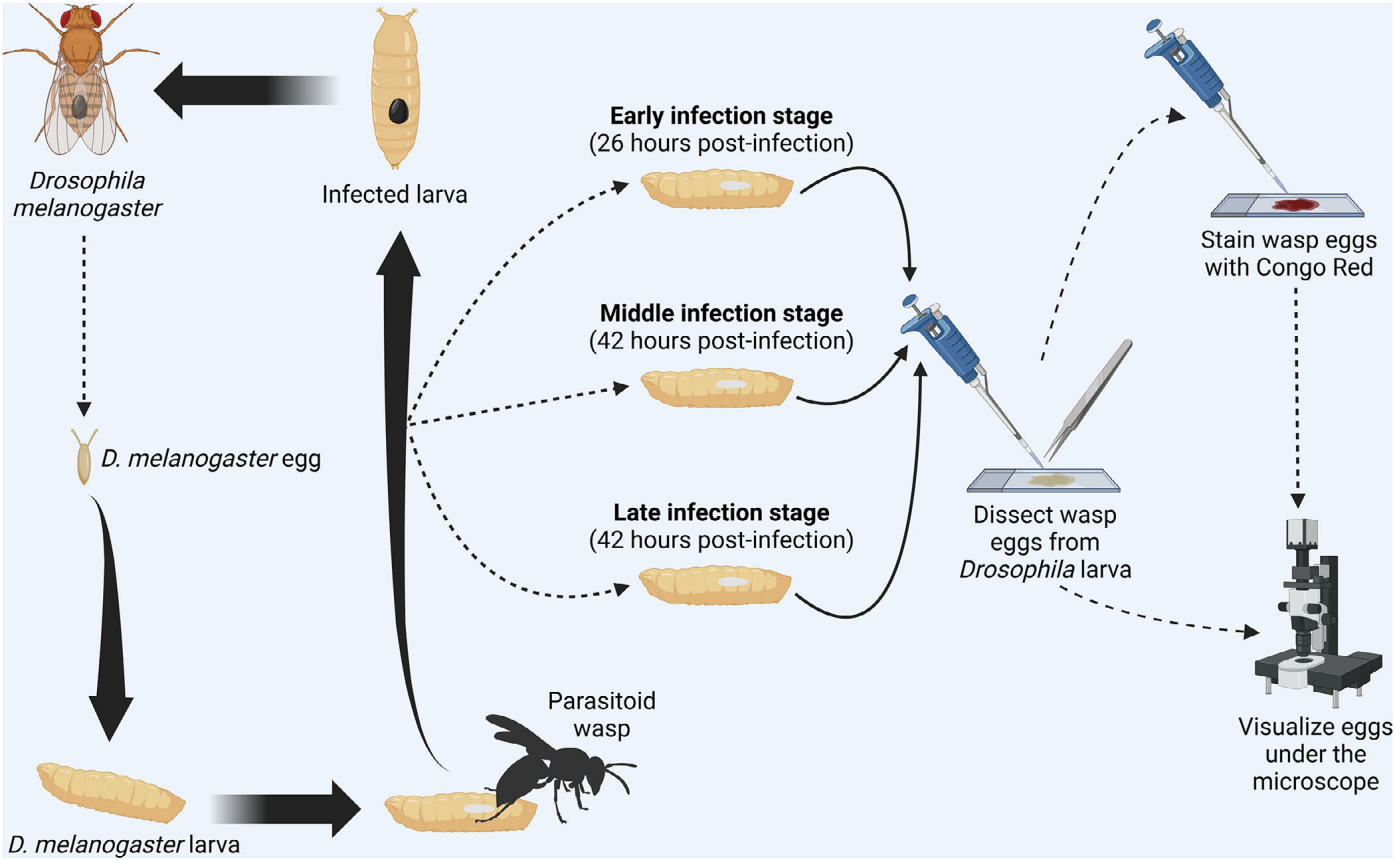# How does amyloid β contribute to healthy immune function in *Drosophila melanogaster*?

**DOI:** 10.1002/alz70855_101820

**Published:** 2025-12-23

**Authors:** Kristen Snitchler, Ashley L Waring, Emma Hartness, Nathan Mortimer

**Affiliations:** ^1^ Oregon State University, Corvallis, OR, USA; ^2^ Illinois State University, Normal, IL, USA

## Abstract

**Background:**

Alzheimer's disease (AD) is a leading cause of death in the United States; as the population continues expanding, so will the number of cases. New drugs targeting amyloid oligomers have been approved to treat AD, but some patients experience serious adverse effects such as swelling and bleeding in the brain, or limited reversal of cognitive impairments. To limit these, must improve our understanding of amyloid β (Aβ) in healthy individuals. We hypothesize that amyloid β acts as an opsonin to direct immune activity to sites of infection or injury.

**Method:**

To test the contribution of Aβ the amyloid precursor protein (APPL) to the *Drosophila* immune response, we used the GAL4/UAS system to develop dAβ and APPL mutants with altered activity. We developed additional *Drosophila* lines to overexpress *Drosophila* amyloid β (dAβ), and a control line with no amyloid expression (Figure 1). Parasitoid wasps infect the developing larvae, and eggs develop to specific time points relating to infection (Figure 3). At each time point, the wasp egg is dissected from the fly larva to assess immune function and stained with Congo‐red to visualize amyloids. Under polarized light, the stain reacts via birefringence when amyloids are present. We can then detect the localization of dAβ over the course of the infection.

**Result:**

We found that dAβ and sAPPL mutant flies exhibit an impaired immune response and capsule formation in response to infection, with the expression of dAβ and sAPPL rescuing this response (Figure 2). While sampling different time points during infection, we detected birefringence on the surface of the developing wasp egg from our dAβ lines prior to the encapsulation. This same signal was absent from our control samples, indicating that dAβ and immune cell activity co‐localize.

**Conclusion:**

Our data suggests a natural function of Aβ is to act as an opsonin and that its aggregation may activate immune cells responsible for inflammation seen in AD.